# Impact of Pupil-Decentration on Visual and Refractive Outcomes in Myopic Patients Undergoing High Astigmatic PRK Surgery

**DOI:** 10.3390/jcm14072282

**Published:** 2025-03-27

**Authors:** Adir Sommer, Margarita Safir, Waseem Nasser, Dror Ben Ephraim Noyman, Tzahi Sela, Gur Munzer, Igor Kaiserman, Eyal Cohen, Michael Mimouni

**Affiliations:** 1 Department of Ophthalmology, Rambam Health Care Campus, Haifa 3109601, Israel; m_mimouni@rambam.health.gov.il (M.M.); 2 Ruth and Bruce Rappaport Faculty of Medicine, Technion-Israel Institute of Technology, Haifa 32000, Israel; 3 Department of Ophthalmology, Rabin Medical Center, Petah Tikva 4941492, Israel; 4 Sackler School of Medicine, Tel Aviv University, Tel Aviv 6997801, Israel; eyalc@tlvmc.gov.il; 5 Care-Vision Laser Center, Tel Aviv 6407810, Israel; gur@care.co.il (G.M.); 6 Department of Ophthalmology, Barzilai Medical Center, Ashkelon 7830604, Israel; 7 Faculty of Health Sciences, Ben-Gurion University of the Negev, Beer Sheva 8410501, Israel; 8 Division of Ophthalmology, Tel Aviv Sourasky Medical Center, Tel Aviv 6423906, Israel

**Keywords:** pupil decentration, high astigmatism, photorefractive keratectomy (PRK), myopic patients, visual and refractive outcomes

## Abstract

**Background/Objectives**: To compare the visual and refractive outcomes of myopic patients undergoing high astigmatic photorefractive keratectomy (PRK) surgery with and without pupillary decentration of treatment. **Methods**: In this retrospective study, we reviewed the medical records of myopic patients who underwent PRK surgery for high astigmatism (>3 diopters [D]) between January 2013 and December 2023. The patients were divided into two groups based on whether the surgeon applied pupillary decentration during surgery. Preoperative, intraoperative, and postoperative parameters were compared between the groups. Adjustments were made to account for differences in baseline characteristics and intraoperative parameters. **Results**: Overall, the study included 575 eyes from 414 patients, of which 79 eyes were treated with pupil decentration. The pupil-decentered group exhibited significantly preoperative higher myopia (subjective spherical equivalent (SEQ) of −5.30 ± 3.12 D vs. −4.26 ± 2.45 D, *p* < 0.001) and subjective sphere (−3.40 ± 3.13 D vs. −2.31 ± 2.49 D, *p* < 0.001). Visual and refractive outcomes, including uncorrected visual acuity (UCVA) of logMAR 0.11 ± 0.77 vs. 0.09 ± 0.72 (*p* = 0.302), best-corrected visual acuity (BCVA) of 0.07 ± 0.92 vs. 0.07 ± 0.82 (*p* = 0.982), SEQ (−0.33 ± 0.93 vs. −0.19 ± 0.60D, *p* = 0.094), sphere (0.02 ± 0.98 vs. 0.15 ± 0.67 D, *p* = 0.142), cylinder (−0.71 ± 0.48 vs. −0.70 ± 0.55 D, *p* = 0.894), safety index (1.07 ± 0.27 vs. 1.12 ± 0.31, *p* = 0.236), and efficacy index (0.99 ± 0.31 vs. 1.07 ± 0.35, *p* = 0.065), showed no significant differences between the two groups. Postoperative outcomes remained comparable after adjusting for baseline and intraoperative parameters. **Conclusions**: Our findings suggest that, in myopic patients undergoing high-astigmatic PRK surgery, pupil decentration does not lead to superior postoperative visual and refractive outcomes. This highlights that, in this scenario, surgeons can preserve the efficacy and safety of the procedure even without applying pupil decentration.

## 1. Introduction

Photorefractive keratectomy (PRK) is widely used to correct refractive errors, including myopia, hyperopia, and astigmatism. A key factor in the success of this procedure is the accurate alignment and centration of the laser ablation [1]. The optical axis is an imaginary line that passes through the centers of curvature of the eye’s optical surfaces, including the cornea and lens. The pupillary axis is an anatomically defined line that is perpendicular to the corneal surface and passes through the center of the entrance pupil. The visual axis/corneal vertex is the line connecting the fovea to the fixation point, passing through the two nodal points of the eye. The line of sight is the line joining the fixation point and the center of the entrance pupil [2], and for a patient who fixates properly, pupillary centration defines the line of sight. Because the optical and anatomical axes are not perfectly aligned in most eyes, this misalignment can impact on visual acuity and quality, as well as the effectiveness of refractive surgeries. During refractive surgery, the surgeon can choose to perform pupillary decentration, which shifts the ablation towards the visual axis. The surgeon has the discretion to determine the degree of pupillary decentration, selecting a percentage shift ranging from 0% (no shift, meaning the treatment is centered on the pupil) to 100% (a complete shift toward the corneal vertex or visual axis). This decision is typically based on factors such as the patient’s refractive error, astigmatism severity, and the surgeon’s clinical judgment and experience. In cases where no pupillary decentration is performed, the laser ablation is centered directly on the pupil, which is considered the default approach. This method assumes that centering on the pupil provides adequate alignment for effective refractive correction, especially when there are no significant anatomical or optical misalignments that might necessitate a shift toward the visual axis. Misalignment between the corneal vertex and pupillary axis can negatively affect visual and refractive outcomes, leading to residual astigmatism, higher-order aberrations (HOAs), and inaccurate refractive corrections, as well as reducing both visual acuity and overall visual quality [2,3,4,5]. For myopic patients with high astigmatism undergoing PRK surgery, precise centration is particularly crucial, more so than in laser-assisted in situ keratomileusis (LASIK) surgery due to the complex ablation profiles required for correcting higher degrees of astigmatism, resulting in less predictability and a higher association of PRK with HOAs compared to LASIK [6]. Any non-optimal centration can therefore lead to complications such as glare, halos, impaired night vision, and residual refractive errors resulting from under correction, overcorrection, or induced irregular astigmatism [6]. In addition, although more prominent in hyperopic patients, corneal-vertex-centered ablations may need adjustment for angle kappa, the angle between the visual and pupillary axes, to optimize visual outcomes [7].

While numerous studies have investigated the efficacy and safety of corneal vertex-centered versus pupil-centered treatments in myopic patients in general [8,9,10,11], few have specifically addressed the role of pupil decentration in patients with high astigmatism [1]. Given the lack of data in the published literature regarding the optimal ablation centration technique for patients with high astigmatism, the purpose of the current study was to compare visual and refractive outcomes in myopic patients with high astigmatism undergoing PRK surgery using pupil-decentration versus no-pupil-decentration treatment, utilizing data from a large patient database.

## 2. Materials and Methods

All data for the study were collected and analyzed in accordance with the policies and procedures of the Institutional Review Board of the Barzilai Medical Center (Ashkelon, Israel) and the tenets of the Declaration of Helsinki.

### 2.1. Study Participants

This retrospective study included patients who underwent their first PRK surgery between January 2013 and December 2023 at Care-Vision Laser Centers, Tel-Aviv, Israel. Inclusion criteria included patients aged 17 to 60, a stable refraction for at least 12 months, intraocular pressure lower than 21 mmHg, and a contact-lenses-free period of more than two weeks for rigid lenses and over three days for soft lenses. The exclusion criteria included patients whose worst-seeing eye had a best-corrected visual acuity (BCVA) of less than logMAR 0.4, patients not targeted for emmetropia, eyes treated for monovision, and patients without postoperative data within a one-year range. High astigmatism was defined as having a cylinder of more than 3 diopters, and myopia was defined as having a manifest refraction spherical equivalent (SEQ) ≤ −0.5 diopters. For analysis purposes, patients were divided into two groups based on whether the surgeon applied pupillary decentration during surgery.

### 2.2. Data Collection

The medical files of all eligible patients were reviewed, and the following demographic and preoperative information was extracted: age, eye (right/left), pachymetry, mean, minimal, and maximal keratometric power (based on keratometry [K] of the Sirius Scheimpflug Tomography (Costruzione Strumenti Oftalmici, Firenze, Italy), uncorrected visual acuity (UCVA), BCVA, spherical equivalent (SEQ), sphere, cylinder power, and axis. The following intraoperative data were extracted: treatment parameters—sphere and cylinder, and maximum ablation depth. Decimal scores were converted to logMAR using the formula logMAR = −log(decimal acuity).

### 2.3. Main Outcome Measures

The main outcome measures were recorded at the last follow-up visit, within the range of three months to one year after surgery. These measures included UCVA, BCVA, subjective SEQ, subjective sphere, subjective cylinder, safety index (postoperative BCVA/preoperative BCVA), and efficacy index (postoperative UCVA/preoperative BCVA). If post-operative data on subjective refraction were available, they were included for analysis as well. In cases where subjective refraction was not available, autorefraction was used instead. HOAs were not recorded in the follow-up meetings.

### 2.4. Surgical Technique

During surgery, one drop of a topical anesthetic (benoxinate hydrochloride 0.4%) was instilled in the conjunctival fornix, after which a lid speculum was inserted. Epithelial removal was performed via alcohol-assisted PRK (20% ethyl alcohol placed on the cornea for 15 s). Following epithelial removal, the WaveLight Allegretto 200 Hz excimer laser (Alcon Laboratories, Fort Worth, TX, USA) was used for wave front optimized stromal ablation under constant eye tracking. The ablation zone was composed of an optic zone of 6.0–7.0 mm and a transition zone of 2.0–3.0 mm. Following excimer ablation, all PRK cases were treated with a 0.02% mitomycin C-soaked sponge placed on the stroma for 30–40, 50–60, or 60–70 s (<6, 6–8, or >8 D of SEQ, respectively). After rinsing the mitomycin, a contact lens was placed. Following surgery, patients were prescribed moxifloxacin 0.5% (4 times a day), dexamethasone 0.1% (4 times a day), and artificial tears without preservatives (4 times a day). Surgery was performed by multiple surgeons using a standard protocol at one surgical center. Patients were routinely examined on 1 day; 1 week; and 1, 3 months postoperatively, and later if necessary. Patients were encouraged to return for examination if vision deteriorated at any time after surgery, and laser enhancement procedures were offered free of charge after surgery whenever needed and after a 6-month period of stable refraction.

### 2.5. Statistical Analysis

The data were analyzed using IBM^®^ SPSS^®^ Statistics software (version 27). An independent samples *t*-test was used to compare the means of the variables. To compare the outcomes between the groups while controlling for potential confounders, a multivariate regression analysis was conducted. The model was adjusted for any preoperative and intraoperative factors that showed statistically significant differences between the parameters. Accordingly, adjustment accounted for differences in baseline (subjective SEQ and cylinder) and intraoperative parameters (treated sphere and cylinder, and maximum ablation depth) (*p* < 0.05 for all). Standardized graphs illustrating the refractive outcomes were generated using the mEYEstro Software (version 2.4) [12]. In all analyses, a two-sided *p*-value of less than 0.05 was considered statistically significant. All means were reported with their corresponding standard deviations (SDs). This work made use of OpenAI’s ChatGPT 4o for proofreading and syntax correction of the results paragraphs in the preparation of the manuscript. The outputs were thoroughly reviewed, revised, and edited by the authors as needed. The authors take full responsibility for the final content of the publication.

## 3. Results

Overall, 575 eyes of 414 patients were included. The mean age was 27.0 ± 7.9 years, and 79 eyes (13.7%) were treated with pupillary decentration.

### 3.1. Preoperative Comparison of Patients Undergoing Pupillary Decentration Treatment vs. Pupil-Centered Ablation

Table 1 compares preoperative baseline variables between patients who underwent pupil-decentered treatment (corneal-vertex-centered) and those who did not (pupillary-centered). There were no significant differences in age (26.7 ± 8.0 vs. 27.0 ± 7.9 years, *p* = 0.691), gender distribution (60.7% vs. 56.6% male, *p* = 0.492), or operated side (48.1% vs. 47.3% right eye, *p* = 0.905). Pachymetry measurements were similar between the groups (534.48 ± 34.77 μm vs. 528.90 ± 31.28 μm, *p* = 0.148), as the average, minimal, and maximal keratometry values (average keratometry was 44.22 ± 1.72 D versus 44.14 ± 1.65 D, minimal keratometry was 42.64 ± 1.76 D versus 42.47 ± 1.64 D, and maximal keratometry was 45.79 ± 1.75 D versus 45.82 ± 1.73 D, respectively, *p* > 0.05 for all). Visual acuity measures, including UCVA (logAMR 1.10 ± 1.00 vs. 0.96 ± 0.89, *p* = 0.081) and BCVA (logMAR 0.09 ± 0.87 vs. 0.11 ± 0.89, *p* = 0.074), did not differ significantly between groups. Similarly, refractive measurements such as subjective cylinder (−3.81 ± 0.63 D vs. −3.89 ± 0.68 D, *p* = 0.278) and subjective axis (92.15 ± 72.08° vs. 96.99 ± 76.13°, *p* = 0.598) were not significantly different. However, the pupil-decentration group exhibited significantly higher myopia in subjective SEQ (−5.30 ± 3.12 D vs. −4.26 ± 2.45 D, *p* < 0.001) and subjective sphere (−3.40 ± 3.13 D vs. −2.31 ± 2.49 D, *p* < 0.001).

### 3.2. Intraoperative Parameters of the Pupillary Decentration and No Pupillary Decentration Groups

As seen in Table 2, the pupil decentration group exhibited a significantly more negative treated sphere (−3.92 ± 2.71 D vs. −2.61 ± 2.38 D, *p* < 0.001) and a less negative treated cylinder (−3.24 ± 0.53 D vs. −3.49 ± 0.90 D, *p* = 0.015). Additionally, the maximum ablation depth was greater (101.78 ± 34.22 µm vs. 88.12 ± 52.51 µm, *p* = 0.026).

### 3.3. Postoperative Visual and Refractive Results of the Pupillary Decentration and No Pupillary Decentration Groups

**Figure 1:**
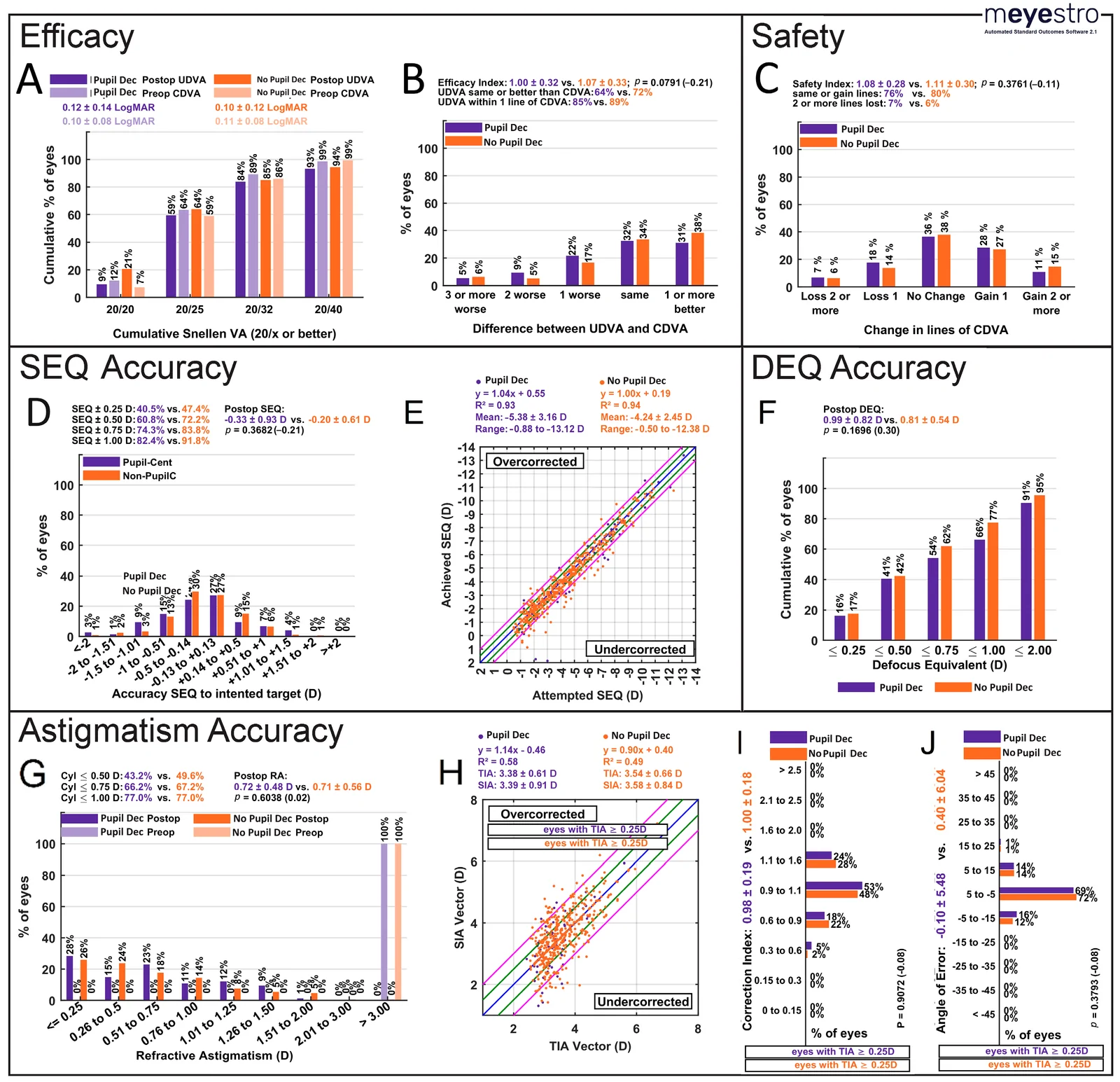
Outcome comparison of pupillary decentration and no pupillary decentration groups. Comparison of visual and refractive outcomes of pupil decentration and no pupil decentration groups: Efficacy: (**A**) cumulative uncorrected (UDVA) and corrected visual acuity (CDVA), (**B**) difference between UDVA and CDVA. Safety: (**C**) change in line of CDVA. Accuracy: (**D**) spherical equivalent (SEQ) to intended target, (**E**) attempted vs. achieved SEQ, (**F**) defocus equivalent (DEQ) accuracy, (**G**) refractive astigmatism accuracy, (**H**) target-induced astigmatism vs. surgically-induced astigmatism, (**I**) correction index histogram, (**J**) angle of error histogram. Percent proportions, means, standard deviations, Cohen’s d effect sizes, and *p*-values are calculated and displayed on each graph. Both groups demonstrated comparable refractive and visual outcomes as well as comparable safety and efficacy indices.

Table 3 compares visual and refractive outcomes between the pupil decentration and no pupil decentration groups. There were no significant differences between the groups in follow-up time (138.2 ± 57.3 days vs. 149.9 ± 66.4 days, *p* = 0.139). Visual acuity outcomes were similar, with UCVA of logMAR 0.11 ± 0.77 vs. 0.09 ± 0.72 (*p* = 0.302) and subjective BCVA of 0.07 ± 0.92 vs. 0.07 ± 0.82 (*p* = 0.982). Refractive measurements also did not differ significantly, including subjective SEQ of −0.33 ± 0.93 D vs. −0.19 ± 0.60 D (*p* = 0.094), subjective sphere of 0.02 ± 0.98 D vs. 0.15 ± 0.67 D (*p* = 0.142), and subjective cylinder of −0.71 ± 0.48 D vs. −0.70 ± 0.55 D (*p* = 0.894). Additionally, the safety index was comparable between groups (1.07 ± 0.27 vs. 1.12 ± 0.31, *p* = 0.236), as was the efficacy index (0.99 ± 0.31 vs. 1.07 ± 0.35, *p* = 0.065). Standardized graphs comparing pupillary decentration and no pupillary decentration groups are provided in Figure 1.

### 3.4. Comparison of Pupillary Decentration and No Pupillary Decentration Groups After Adjustment for Potential Confounders

After adjusting for differences in baseline (subjective SEQ and cylinder) and intraoperative parameters (treated sphere and cylinder, and maximum ablation depth) (*p* < 0.05 for all), further analysis did not demonstrate significant differences between the pupil decentration and no pupil groups in postoperative outcomes. UCVA and BCVA did not differ significantly (0.14 ± 0.68 vs. 0.11 ± 0.70, *p* = 0.159, and 0.06 ± 0.96 vs. 0.07 ± 085, *p* = 0.761, respectively). Refractive measurements also did not differ significantly, including subjective SEQ of −0.32 ± 0.56 D vs. −0.17 ± 0.53 D (*p* = 0.132), subjective sphere of 0.007 ± 0.62 D vs. 0.17 ± 0.64 D (*p* = 0.147), and subjective cylinder of −0.70 ± 0.61 D vs. −0.65 ± 0.47 D (*p* = 0.687). Additionally, the safety index was comparable between the groups (1.12 ± 0.24 vs. 1.10 ± 0.29, *p* = 0.709), as was the efficacy index (0.94 ± 0.33 vs. 1.01 ± 0.35, *p* = 0.305) (Table 4).

## 4. Discussion

This study aimed to assess the visual and refractive outcomes of high astigmatic PRK surgery performed with and without pupillary decentration in myopic patients. Our findings indicate that pupillary decentration does not provide superior postoperative outcomes compared to no pupillary decentration treatments in terms of UCVA, BCVA, or refractive measures such as SEQ and postoperative astigmatism. Both groups exhibited comparable safety and efficacy indices, indicating that pupillary decentration did not enhance the overall success of the procedure.

The optical axis and anatomical axis of vision play important roles in understanding and treating myopia in refractive surgeries. Several studies have explored the differences between these axes and their implications for vision correction, particularly in the context of myopia treatment [2,13]. While pupil-centered ablation (i.e., no pupillary decentration applied) is currently the most commonly used centration method [10], there remains ongoing debate about which centration approach leads to better visual and refractive outcomes. Many physicians base their decision to apply pupillary decentration on their personal experience and clinical judgment, leading to variability in practice [2].

The implementation of pupillary decentration in patients with high astigmatism varies among surgeons in our institution. Some physicians do not apply pupillary decentration in cases without astigmatism or with low cylindrical power (the threshold varies by physician). However, there is generally a consensus to apply a certain percentage of pupillary decentration in patients with high astigmatism, with the degree of decentration (0–100%) depending on the physician’s preferences and experience.

In high astigmatic eyes, precise centration of the laser ablation is critical due to the complex and irregular corneal curvature that characterizes these cases. High astigmatism involves significant differences in refractive power across various meridians, making the optical system more sensitive to even minor decentrations. When the ablation is centered on the pupil, which is inherently unstable and subject to changes with varying lighting conditions and accommodation, the risk of misalignment increases. Such misalignment can disproportionately induce HOAs, including coma and spherical aberrations, and may lead to visual disturbances like glare, halos, and reduced contrast sensitivity [14].

By contrast, centering the ablation on the corneal vertex provides a more stable and anatomically consistent reference point that closely approximates the true optical axis. This approach minimizes abrupt curvature transitions between the treated and untreated corneal zones, preserving the natural corneal shape and reducing the potential for decentration-induced aberrations. In high astigmatic patients, where even small deviations can have a pronounced impact on postoperative visual quality, shifting the ablation center toward the corneal vertex via pupillary decentration becomes essential for optimizing outcomes, whereas in patients with low astigmatism, the impact of such variability is less significant, diminishing the necessity for decentration.

Our findings are consistent with previous studies on myopic patients undergoing refractive surgery, which did not demonstrate significant advantages of one centration method over another—for example, pupillary centration compared to corneal vertex centering [8,9,14,15,16]. Notably, a study by Rabine et al. showed that myopic patients undergoing wavefront optimized LASIK or PRK demonstrated similar outcomes with both pupil-centered and corneal-vertex-centered ablation treatments [10]. In their study, both LASIK and PRK patients did not differ in postoperative efficacy and safety indices, nor in residual astigmatism. In a meta-analysis by Zhang et al., both corneal-vertex and pupillary-centered approaches demonstrated comparable refractive outcomes in myopic patients, but the corneal-vertex method exhibited less postoperative HOAs, mainly coma aberrations [17]. According to several other studies, pupil-centered ablation can influence HOAs and overall visual quality, as the ablation center tends to deviate more from the visual axis in pupil-centered procedures compared to other techniques, such as centration on the coaxially sighted corneal light reflex (CSCLR) [7,17]. This greater deviation, along with potentially increased ablation depth, may contribute to the higher induction of HOAs, which can negatively impact the patient’s visual quality. Moreover, since the alignment of the ablation zone relative to the pupil center and corneal apex can impact visual outcomes, proper centration is crucial for achieving optimal results, particularly in patients with high astigmatism [18]. Nevertheless, since HOAs generally lead to a decrease in the quality of vision but not in visual acuity, even if there were theoretically more HOAs in the pupil-centration group, this would not result in a difference in visual acuity, as observed in our study. Furthermore, since angle kappa is typically larger in hyperopic patients and less significant in myopic patients, those undergoing myopic treatment might be less sensitive to centration errors due to not applying pupillary decentration. In addition to the above, when making a decision regarding the preferred method of centration, it should also be taken into account that, according to studies, pupil center may also shift significantly with changing illumination, making it less reliable for centration [19,20].

Overall, the findings suggest that surgeons can perform high astigmatic PRK surgery without necessarily adhering to pupillary decentration while maintaining both safety and efficacy. This flexibility may simplify the surgical process and reduce the need for meticulous alignment, while still achieving excellent visual and refractive results.

Nevertheless, our study has limitations to consider. The retrospective nature of the study may introduce selection bias, and the follow-up period, while sufficient to assess short- to mid-term outcomes, may not capture long-term refractive stability. Moreover, during the postoperative follow-up, HOAs were not recorded, making it impossible to assess their potential impact on patients’ postoperative visual quality in our study population. This is a major limitation as most current studies discuss the advantages and disadvantages of different centration methods, primarily focusing on their impact on the occurrence of HOAs after surgery. We only recorded whether astigmatism was present or not, without additional details on the matter. Additionally, patients who did not undergo pupil-centered treatment may have been operated on by different surgeons than those who performed pupil-centered treatment. Furthermore, there was no documentation on the percentage of decentration performed and the extent of the shift from the pupil toward the visual axis. In cases where no shift was implemented during the procedure, it remains uncertain as to whether this decision was influenced by technical limitations of the laser system or specific clinical factors. It is possible that the laser system was unable to consistently or accurately track the patient’s eye movements. Alternatively, the decision could have been influenced by other factors, such as the absence of significant astigmatism. These uncertainties highlight the need for improved documentation and a better understanding of the factors influencing surgical choices in such scenarios. However, even when accounting for potential bias arising from differences in astigmatism among patients, which could influence the surgeon’s decision on whether to apply pupillary decentration, the results, after adjusting for confounding factors, including astigmatism, still demonstrated no significant differences in outcomes between the groups. Future prospective studies with extended follow-up periods are recommended to confirm the durability of these outcomes. Moreover, further research is warranted to explore whether advanced technologies, such as advanced eye-tracking systems [21], can further enhance the precision and effectiveness of pupil decentration in high astigmatic PRK treatments.

In conclusion, this study suggests that in myopic patients undergoing high-astigmatic PRK surgery, pupil decentration does not lead to superior postoperative visual and refractive outcomes. This highlights that, in this scenario, surgeons will not compromise the efficacy or safety of the procedure if they choose not to perform pupil decentration.

## Figures and Tables

**Table 1:** Comparison of preoperative parameters.

Parameter Name	Pupil Decentration (*n* = 79)	No Pupil Decentration (*n* = 496)	*p*-Value
Age (years)	26.7 ± 8.0	27.0 ± 7.9	0.691
Gender (% male)	60.7	56.6	0.492
Side (% right)	48.1	47.3	0.905
Pachymetry (μm)	534.48 ± 34.77	528.90 ± 31.28	0.148
Average keratometry (D)	44.22 ± 1.72	44.14 ± 1.65	0.707
Minimal keratometry (D)	42.64 ± 1.76	42.47 ± 1.64	0.382
Maximal Keratometry (D)	45.79 ± 1.75	45.82 ± 1.73	0.909
UCVA (logMAR)	1.10 ± 1.00	0.96 ± 0.89	0.081
Subjective SEQ (D)	−5.30 ± 3.12	−4.26 ± 2.45	<0.001
Subjective sphere (D)	−3.40 ± 3.13	−2.31 ± 2.49	<0.001
Subjective cylinder (D)	−3.81 ± 0.63	−3.89 ± 0.68	0.278
Subjective axis (degrees)	92.15 ± 72.08	96.99 ± 76.13	0.598
Subjective BCVA (logMAR)	0.09 ± 0.89	0.11 ± 0.89	0.074

Comparison of preoperative parameters between patients undergoing PRK surgery with and without pupil decentration. UCVA, uncorrected visual acuity; BCVA, best corrected visual acuity; SEQ, spheric equivalent; D, diopters.

**Table 2:** Comparison of intraoperative parameters.

Parameter Name	Pupil Decentration	No Pupil Decentration	*p*-Value
Treated sphere (D)	−3.92 ± 2.71	−2.61 ± 2.38	<0.001
Treated cylinder (D)	−3.24 ± 0.53	−3.49 ± 0.90	0.015
Maximum ablation depth (µm)	101.78 ± 34.22	88.12 ± 52.51	0.026

Comparison of intraoperative parameters between patients undergoing PRK surgery with and without pupil decentration. D, diopters.

**Table 3:** Comparison of visual and refractive outcomes.

Parameter Name	Pupil Decentration	No Pupil Decentration	*p*-Value
Follow-up time (days)	138.2 ± 57.3	149.9 ± 66.4	0.139
UCVA (logMAR)	0.11 ± 0.77	0.09 ± 0.72	0.302
Subjective SEQ (D)	−0.33 ± 0.93	−0.19 ± 0.60	0.094
Subjective sphere (D)	0.02 ± 0.98	0.15 ± 0.67	0.142
Subjective cylinder (D)	−0.71 ± 0.48	−0.70 ± 0.55	0.894
Subjective BCVA (logMAR)	0.07 ± 0.92	0.07 ± 0.82	0.982
Safety index	1.07 ± 0.27	1.12 ± 0.31	0.236
Efficacy index	0.99 ± 0.31	1.07 ± 0.35	0.065

Comparison of postoperative visual and refractive outcomes between patients undergoing PRK surgery with and without pupil decentration. UCVA, uncorrected visual acuity; BCVA, best corrected visual acuity; SEQ, spheric equivalent; D, diopters.

**Table 4:** Comparison of visual and refractive outcomes after adjustment for pre- and intra-operative variables.

Parameter Name	Pupil Decentration	No Pupil Decentration	*p*-Value
UCVA (logMAR)	0.14 ± 0.68	0.11 ± 0.70	0.159
Subjective SEQ (D)	−0.32 ± 0.56	−0.17 ± 0.53	0.132
Subjective sphere (D)	0.007 ± 0.62	0.17 ± 0.64	0.147
Subjective cylinder (D)	−0.70 ± 0.61	−0.65 ± 0.47	0.687
Subjective BCVA (logMAR)	0.06 ± 0.96	0.07 ± 0.85	0.761
Safety index	1.12 ± 0.24	1.10 ± 0.29	0.709
Efficacy index	0.94 ± 0.33	1.01 ± 0.35	0.305

Adjusted comparison of postoperative visual and refractive outcomes between patients undergoing PRK surgery with and without pupil decentration. UCVA, uncorrected visual acuity; BCVA, best corrected visual acuity; SEQ, spheric equivalent; D, diopters.

## Data Availability

The datasets presented in this article are not readily available due to commercialization restrictions. Requests to access the datasets should be directed to sela@care.co.il.
