# Correction: Sommer et al. Impact of Pupil-Decentration on Visual and Refractive Outcomes in Myopic Patients Undergoing High Astigmatic PRK Surgery. *J. Clin. Med.* 2025, *14*, 2282

**DOI:** 10.3390/jcm15176644

**Published:** 2026-08-28

**Authors:** Adir Sommer, Margarita Safir, Waseem Nasser, Dror Ben Ephraim Noyman, Tzahi Sela, Gur Munzer, Igor Kaiserman, Eyal Cohen, Michael Mimouni

**Affiliations:** 1 Department of Ophthalmology, Rambam Health Care Campus, Haifa 3109601, Israelm_mimouni@rambam.health.gov.il (M.M.); 2 Ruth and Bruce Rappaport Faculty of Medicine, Technion-Israel Institute of Technology, Haifa 32000, Israel; 3 Department of Ophthalmology, Rabin Medical Center, Petah Tikva 4941492, Israel; 4 Sackler School of Medicine, Tel Aviv University, Tel Aviv 6997801, Israel; eyalc@tlvmc.gov.il; 5 Care-Vision Laser Center, Tel Aviv 6407810, Israelgur@care.co.il (G.M.);; 6 Department of Ophthalmology, Barzilai Medical Center, Ashkelon 7830604, Israel; 7 Faculty of Health Sciences, Ben-Gurion University of the Negev, Beer Sheva 8410501, Israel; 8 Division of Ophthalmology, Tel Aviv Sourasky Medical Center, Tel Aviv 6423906, Israel

## Missing Citation

In the original publication [1], “Gauvin, M.; Wallerstein, A. mEYEstro software: An automatic tool for standardized refractive surgery outcomes reporting. *BMC Ophthalmol.* **2023**, *23*, 171. https://doi.org/10.1186/s12886-023-02904-6” was not cited. The citation has now been inserted into Section 2. Materials and Methods, Section 2.5. Statistical Analysis, paragraph one, and should read:

The data were analyzed using IBM^®^ SPSS^®^ Statistics software (version 27). An independent samples *t*-test was used to compare the means of the variables. To compare the outcomes between the groups while controlling for potential confounders, a multivariate regression analysis was conducted. The model was adjusted for any preoperative and intraoperative factors that showed statistically significant differences between the parameters. Accordingly, adjustment accounted for differences in baseline (subjective SEQ and cylinder) and intraoperative parameters (treated sphere and cylinder, and maximum ablation depth) (*p* < 0.05 for all). Standardized graphs illustrating the refractive outcomes were generated using the mEYEstro Software (version 2.4) [12]. In all analyses, a two-sided *p*-value of less than 0.05 was considered statistically significant. All means were reported with their corresponding standard deviations (SDs).

## Reference

12. Gauvin, M.; Wallerstein, A. mEYEstro software: An automatic tool for standardized refractive surgery outcomes reporting. *BMC Ophthalmol.* **2023**, *23*, 171. https://doi.org/10.1186/s12886-023-02904-6.

With this correction, the order of some references has been adjusted accordingly. The authors state that the scientific conclusions are unaffected. This correction was approved by the Academic Editor. The original publication has also been updated.
